# Reply to the Letter to the Editor by A. Schwelm and S. Neuhauser: “Detection of Ribosomal DNA Sequence Polymorphisms in the Protist *Plasmodiophora brassicae* for the Identification of Geographical Isolates”

**DOI:** 10.3390/ijms18071455

**Published:** 2017-07-06

**Authors:** Rawnak Laila, Jong-In Park, Arif Hasan Khan Robin, Kiwoung Yang, Gyung Ja Choi, Ill-Sup Nou

**Affiliations:** 1 Department of Horticulture, Sunchon National University, Suncheon 540-950, Korea; rawnak.2010@gmail.com (R.L.); jipark@sunchon.ac.kr (J.-I.P.); gpb21bau@gmail.com (A.H.K.R.); ykw7685@naver.com (K.Y.); 2 Department of Genetics and Plant Breeding, Bangladesh Agricultural University, Mymensingh-2202, Bangladesh; 3 Center for Eco-friendly New Materials, Korea Research Institute of Chemical Technology, Daejeon 34114, Korea; kjchoi@krict.re.kr

The authors of Laila et al. [1] would like thank to the readers (A. Schwelm and S. Neuhauser) for submitting a letter requesting the authors to correct ribosomal DNA (rDNA) sequences of 11 Korean *Plasmodiophora bassicae* isolates at the 3′-end. After re-sequencing 2069 bp large subunit (LSU) sequences at the 3′-end, the authors confirm that in the main text of our original paper [1]: Figures 4–6 are redundant. In the supplementary materials of our original paper [1]: Table S1, Figures S4–S6 and S8 and Probes 5516, 6754 and 6479 in Figure S7 are redundant. 

Here, we have corrected rDNA sequences of 11 Korean *P. bassicae* isolates and submitted the revised sequences to NCBI. The up-to-date accession numbers of rDNA sequences are given in Table 1.

The updated rDNA sequences of *P. brassicae* of Korean isolates now have over 98% sequence identity with reference sequence KX011115. Korean isolates Yeoncheon, Daejon, Haenam2, Gangneung2, Haenam1 and Geumsan have identical rDNA sequences. These six isolates have less than 1% sequence dissimilarities with another five isolates.

The phylogenetic tree grouped rDNA sequences of both Korean and reference isolates, reported by Schwelm et al. [2], of *P. brassicae* in the same cluster as shown in Figure 1. Thus, the phylogenetic tree separated rDNA sequences of Korean and reference isolates from other cercozoans. Strikingly, the Japanese NGY isolate AB526853 [3] clustered with non-parasitic Glissomonadida, *Heteromita globosa*. 

We previously used the rDNA sequences of the Japanese NGY isolate, accession number AB526843 [3], as a reference for designing primers to obtain rDNA sequences of 11 Korean *P. bassicae* isolates. There were no complete published rDNA sequences except AB526843 when we started sequencing and obtained rDNA sequences of selected Korean *P. bassicae* isolates. In a recent article, it was reported that rDNA sequences of NGY isolate were chimeric in nature between *P. brassicae* and non-parasitic Glissomonadida [2]. We became aware of this issue after our article was published [1]. 

We have now, therefore, designed primers based on the following two sequences, KX011115 and KX011135, published by Schwelm et al. [2] to correct sequences of LSU of 11 Korean *P. bassicae* isolates that mismatched with recently reported sequences (Table 2). The procedure of constructing the phylogenetic tree has been described in Laila et al. [1].

In conclusion, Among 11 Korean *P. brassicae* isolates Yeoncheon, Daejon, Haenam2, Gangneung2, Haenam1 and Geumsan had identical rDNA sequences. The other five isolates had less than 1% sequence dissimilarities with those six isolates. The rDNA sequences of 11 Korean *P. brassicae* including LSU had 98% identity with the reference rDNA sequences of European isolates KX011115 and KX011135. 

## Figures and Tables

**Figure 1:**
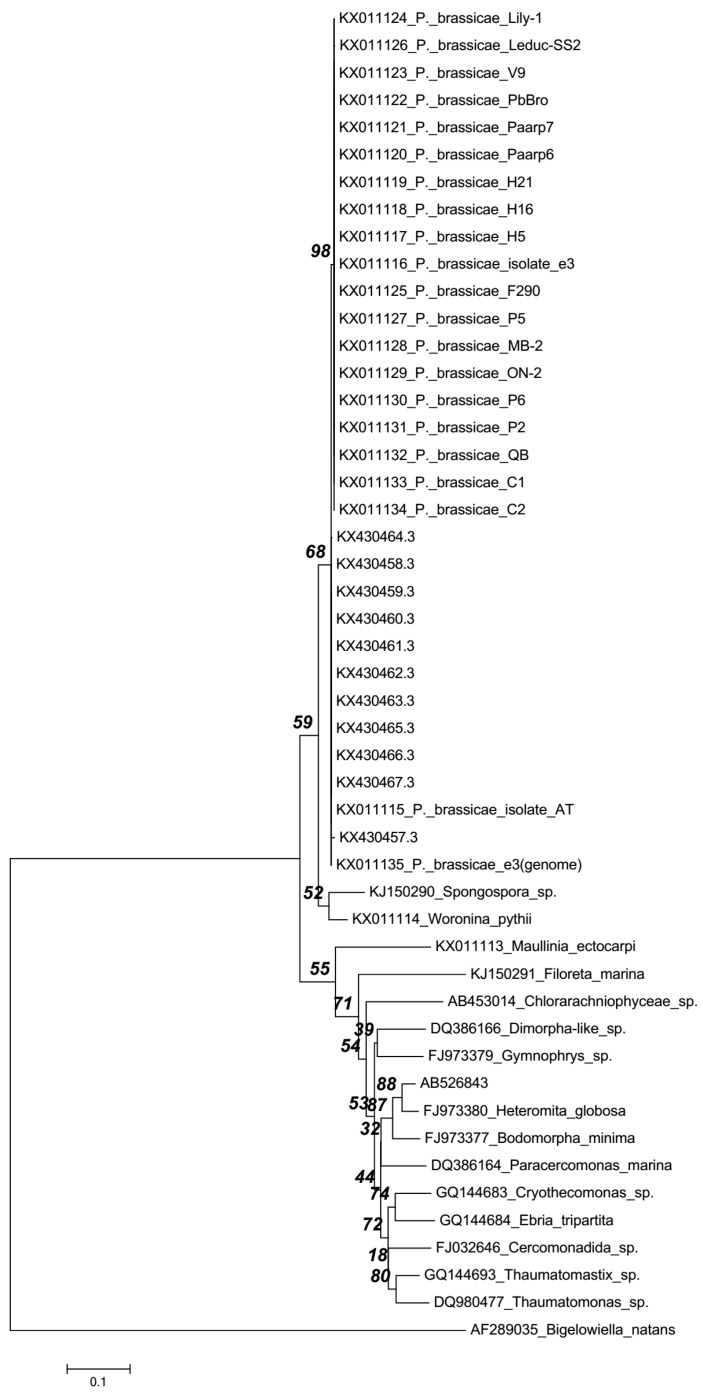
Phylogenetic tree constructed following the Neighbor-joining method and Maximum Composite Likelihood model in Mega6.06 software between the 11 Korean field isolates and the reference isolates of *P. brassicae* based on variations in the nucleotide sequences of rDNA. Complete rDNA, if available, or LSU sequences were used.

**Table 1:** The up-to-date GenBank accession numbers of ribosomal DNA sequences of 11 Korean *Plasmodiophora bassicae* isolates.

Different Geographic Isolates	Accession No.	Sequence Length (bp)	Sequence Encoding
Gangneung1	KX430457.3	7421	Partial ribosomal RNA, both small and large subunits
Yeoncheon	KX430458.3	7421	
Daejon	KX430459.3	7425	
Haenam2	KX430460.3	7426	
Seosan	KX430461.3	7423	
Pyeongchang	KX430462.3	7424	
Gangneung2	KX430463.3	7421	
Haenam1	KX430464.3	7425	
Hoengseong	KX430465.3	7425	
Geumsan	KX430466.3	7424	
Goesan	KX430467.3	7424	

**Table 2:** List of primer sequences used for cloning and sequencing the large subunit of rDNA of target region of *P. brassicae*.

Primer Name	Target Region	Forward Primer	Reverse Primer	Product Size (bp)
LSU1	5391–5671	GAACCAGGACGTGGATATTGTATGG	CCGACTTCCCTTACCTACATTGTT	281
LSU2	5648–6639	CATTGATTCTCCCGCTGGGCCC	GCTGTGGTTTCGCTAGATAGTAGA	1185
LSU3	6549–7459	GGAGTTCGAACAGCTCTTAAGGTA	GTATAGTATGATGCCCGACCCATT	911
